# MicroRNA Gene Expression Signature Driven by *miR-9* Overexpression in Ovarian Clear Cell Carcinoma

**DOI:** 10.1371/journal.pone.0162584

**Published:** 2016-09-09

**Authors:** Nozomu Yanaihara, Yukiko Noguchi, Misato Saito, Masataka Takenaka, Satoshi Takakura, Kyosuke Yamada, Aikou Okamoto

**Affiliations:** 1 Department of Obstetrics and Gynecology, The Jikei University School of Medicine, Tokyo, Japan; 2 Department of Obstetrics and Gynecology, Dokkyo Medical University Koshigaya Hospital, Saitama, Japan; The University of Hong Kong, CHINA

## Abstract

Previous studies have identified microRNA (miRNA) involvement in human cancers. This study aimed to elucidate potential clinical and biological associations of ovarian cancer-related miRNA gene expression profiles in high-grade serous carcinoma (HGSC) and ovarian clear cell carcinoma (OCCC). Accordingly, we investigated 27 patients with ovarian cancer (12 HGSC and 15 OCCC cases) using quantitative real-time reverse transcription polymerase chain reaction to determine the cancer-related miRNA expressions. Gene Cluster 3.0 was used for hierarchical clustering analysis, and differentially expressed miRNAs between HGSC and OCCC were identified by the class comparison analysis using BRB-ArrayTools. An unsupervised hierarchical clustering analysis identified two distinct miRNA expression clusters, with histological subtype-related significant differences in the associations between clusters and clinicopathological features. A comparison of miRNA expression in HGSCs and OCCCs identified five miRNAs (*miR-132*, *miR-9*, *miR-126*, *miR-34a*, and *miR-21*), with OCCCs demonstrating a statistically higher expression. Further investigation of the biological significance of *miR-9* overexpression in OCCC revealed that *miR-9* inhibition reduced the cell invasion ability and upregulated E-cadherin expression. Using a luciferase reporter assay, we further demonstrated the direct binding of *miR-9* to E-cadherin. Global cancer-related miRNA expression analysis identified statistically unique profiles that could discriminate ovarian cancer histotypes. In OCCC, *miR-9* overexpression may affect pathogenesis by targeting E-cadherin, thereby inducing an epithelial–mesenchymal transition. Therefore, *miR-9* may be a promising therapeutic target strategy for OCCC.

## Introduction

MicroRNAs (miRNAs) function as tumor suppressors and oncogenes, thereby playing substantial roles in the development and progression of human cancers. Therefore, miRNAs appear to be promising clinical targets in future cancer diagnostic and treatment applications [1, 2]. To date, comprehensive miRNA microarray analyses have identified particular expression signatures of solid tumors, including lung, breast, stomach, prostate, colon, and ovarian tumors [3, 4]. Iorio et al. [5] provided the first report wherein genome-wide miRNA expression profiling elucidated both ovarian cancer- and histotype-specific miRNA expression patterns. Since then, emerging evidences have suggested that miRNAs play considerable roles in ovarian cancer pathogenesis and have potential use as clinical biomarkers of this most lethal gynecological malignancy.

Four major histological subtypes of epithelial ovarian cancer have been described, and the distributions of these subtypes differ among North America, Europe, and Japan [6]. Globally, high-grade serous carcinoma (HGSC) is the most common histological subtype; in Japan, HGSC is followed by ovarian clear cell carcinoma (OCCC) that has a reported prevalence of approximately 25% of all ovarian cancer cases and has unique clinical and biological characteristics. Typically, OCCC exhibits chemoresistance to standard platinum agent-based chemotherapy, which is a characteristic that is associated with poor prognosis. A recent large clinical trial of conventional paclitaxel and carboplatin chemotherapy versus irinotecan hydrochloride plus cisplatin (CPT-P) first-line chemotherapy for OCCC yielded negative results [7]. Therefore, establishing a new therapeutic approach for this disease is clinically critical.

In this study, we sought to elucidate the clinical and biological implications of ovarian cancer-related miRNA gene expression profiles, particularly focusing on HGSC and OCCC. Our findings indicated that these histological ovarian cancer subtypes could be distinguished using miRNA expression signatures. Through in vitro knockdown experiments, we investigated the biological role of *miR-9* overexpression in OCCC and found that *miR-9* could modulate tumor cell invasion by directly targeting and downregulating E-cadherin expression.

## Materials and Methods

### Clinical samples and cell lines

This study involved surgically obtained tumor specimens of primary ovarian cancers that were collected from patients who were treated at the Department of Obstetrics and Gynecology, The Jikei University School of Medicine. The Ethics Review Committee of The Jikei University School of Medicine approved the study protocol (approval number: 14–132, 27–076), and all patients provided written informed consent prior to the study. Tumors were staged according to the International Federation of Gynecology and Obstetrics staging system (1988). Table 1 presents the clinicopathological characteristics of the original 27 cases investigated in this study and 23 additional cases used as a validation data set.

**Table 1:** Clinicopathological Characteristics of Patients with Ovarian Cancer.

Variables	Original cohort	Additional cohort	Total
	n = 27 (%)	n = 23 (%)	n = 50 (%)
**Patient age**			
**≤60 years**	23 (85)	20 (87)	43 (86)
**>60 years**	4 (15)	3 (13)	7 (14)
**FIGO stage**			
**I + II**	18 (67)	11 (48)	29 (58)
**III + IV**	9 (33)	12 (52)	21 (42)
**Histological type**			
**OCCC**	15 (56)	10 (43)	25 (50)
**HGSC**	12 (44)	13 (57)	25 (50)
**LN metastasis (n = 49)**			
**No metastasis**	18 (69)	18 (78)	36 (73)
**Metastasis**	8 (31)	5 (22)	13 (27)
**CA125 (n = 48)**			
**>35**	20 (80)	20 (87)	40 (83)
**≤35**	5 (20)	3 (13)	8 (17)
**Ascitic cytology (n = 49)**			
**Positive**	14 (54)	16 (70)	30 (61)
**Negative**	12 (46)	7 (30)	19 (39)
**Rupture**			
**Without**	7 (26)	5 (22)	12 (24)
**Surgical**	5 (18)	1 (4)	6 (12)
**Ruptured**	15 (56)	17 (74)	32 (64)
**Endometriosis (n = 49)**			
**Positive**	12 (46)	11 (48)	23 (47)
**Negative**	14 (54)	12 (52)	26 (53)
**Thrombosis (n = 45)**			
**Positive**	2 (8)	3 (14)	5 (11)
**Negative**	22 (92)	18 (86)	40 (89)

FIGO, International Federation of Gynecology and Obstetrics; LN, lymph node; OCCC, ovarian clear cell carcinoma; HGSC, high-grade serous carcinoma

In this study, 16 cell lines, including 10 human OCCC cell lines (JHOC-5, JHOC-7, JHOC-9, HAC-2, RMG-I, RMG-II, OVTOKO, OVMANA, OVISE, and ES-2) and six human ovarian non-OCCC cell lines (2008, A2780, SKOV3, OV-1063, MCAS, and Tyk-nu), were used and maintained as previously described [8]. The following researchers kindly provided the cell lines: Dr. D. Aoki (Keio University, Tokyo, Japan), RMG-I and RMG-II; Dr. M. Nishida (Tsukuba University, Tsukuba, Japan), HAC-2; Dr. E. Reed (National Cancer Institute, Bethesda, MD, USA), A2780 (undifferentiated carcinoma); and Dr. S.B. Howell (University of California–San Diego, San Diego, CA, USA), 2008 (serous carcinoma). Three OCCC cell lines (JHOC-5, JHOC-7, and JHOC-9) were obtained from the Riken Bioresource Center (Tsukuba, Japan) and three (OVTOKO, OVMANA, and OVISE) were obtained from the JCRB Cell Bank (Osaka, Japan). SKOV3 (adenocarcinoma), MCAS (mucinous carcinoma), OV-1063 (ovarian metastatic adenocarcinoma), Tyk-nu (undifferentiated carcinoma), and ES-2 were purchased from ATCC (Rockville, MD, USA). JHOC-9 and OVISE cells, which were used for functional analyses, were authenticated by short tandem repeat analysis and were negative for mycoplasma contamination by Cycleave^®^PCR Mycoplasma Detection Kit (Takara Bio Inc., Shiga, Japan).

### Quantitative reverse transcription polymerase chain reaction analysis

Freshly excised surgical specimens containing >80% of cancer cells by cryostat sections were stored in RNA*later*^®^ Solution (Thermo Fisher Scientific, MA, USA) at 4°C for 24 h and subsequently frozen at −80°C prior to RNA isolation with TRIzol^®^ Reagent (Thermo Fisher Scientific). The miRCURY LNA^™^ Universal RT microRNA PCR and Universal cDNA Synthesis kit was used for reverse transcription (RT; EXIQON, Vedbaek, Denmark), and the resulting cDNAs were subjected to quantitative real-time RT-polymerase chain reaction (PCR) analysis of cancer-associated miRNA expressions.

Eighty-eight miRNA expression profiles including 85 cancer-related miRNAs were quantified using Cancer Focus microRNA PCR Panel V1 (EXIQON) and ExiLENT SYBR^®^ Green master mix (EXIQON). In addition, *miR-132*, *miR-9*, *miR-126*, and *miR-34a* expression profiles were analyzed using TaqMan^®^ MicroRNA Assays (Applied Biosystems, Foster City, CA, USA). All PCR reactions were performed in 96-well plates using the StepOnePlus^™^ Real-Time PCR System (Applied Biosystems). *SNORD38B* was used as the endogenous control, and RMG-I miRNA expression profile was set as the reference. miRNA expression was quantified using a comparative method (2^−ΔΔCT^), where CT is the threshold cycle and ΔΔCt = (CT_miRNA_ − CT_SNORD38B_) − (CT_reference_ − CT_SNORD38B_), as previously described [8].

### Transfection assay

JHOC-9 and OVISE cells were seeded into 6-well plates at a density that would yield 50% confluency after 24 h and were subsequently transfected with the mirVana^™^ miRNA inhibitor (Thermo Fisher Scientific) that was specific for *miR-9* (miR-9 inhibitor) or Anti-miR^™^ miRNA Inhibitor Negative Control (anti-miR-NC; Thermo Fisher Scientific) at a final concentration of 100nM. Lipofectamine^®^ RNAiMAX Transfection Reagent (Thermo Fisher Scientific) was used for transfection, according to the manufacturer’s protocol.

### Migration and invasion assays

To assess cell invasion and migration abilities, JHOC-9 and OVISE cells were transfected with either miR-9 inhibitor or anti-miR-NC for 24 h and seeded in the upper chambers of either 24-well Matrigel-coated polyethylene terephthalate membrane inserts (pore size, 8 μm; Corning, Tewksbury, MA, USA) for invasion assay or 24-well Falcon^®^ Cell Culture Insert (pore size, 8 μm; Corning) for migration assay. The bottom chambers were filled with 0.75 ml of either a medium containing 1% fetal bovine serum (FBS) for JHOC-9 or no FBS for OVISE. After a 48-h incubation, filter membranes were fixed with 100% methanol and subjected to Wright–Giemsa staining. Invading and migrating cells were analyzed via microscopy by counting the numbers of cells in three random fields, with a ×100 magnification. This experiment was repeated at least three times, and results are shown as means ± standard deviations (SDs).

### Western blot analysis

Cells were lysed, and proteins were extracted using 1× RIPA buffer; protein concentrations were subsequently determined using the *DC*^™^ Protein Assay (Bio-Rad Laboratories, Hercules, CA, USA). Total proteins were resolved on NuPage 4%–12% Bis-Tris gels (Thermo Fisher Scientific). Proteins were then transferred to membranes using an iBlot^®^ Gel Transfer Device (Thermo Fisher Scientific).

The membranes were incubated with primary antibodies specific for E-cadherin (clone EP700Y; 1:50000 dilution; Abcam, Cambridge, UK), Vimentin (clone H-84; 1:1000; Santa Cruz, Dallas, TX, USA), Fibronectin (clone F1; 1:1000; Abcam), MMP-9 (clone 65-2A4; 1:500; Daiichi Fine Chemical, Toyama, Japan), or β-actin (clone 13E5; 1:1000; Cell Signaling Technology, Danvers, MA, USA) overnight at 4°C. All antibodies were diluted in Tris-buffered saline (TBS) containing 0.1% Tween 20 and 5% bovine serum albumin. Subsequently, blots were incubated with an horseradish peroxidase-conjugated secondary antibody (Cell Signaling Technology; 1:10000) in TBS with 0.1% Tween 20 and 5% nonfat milk for 1 h at room temperature with gentle agitation. Blots were visualized using ImmunoStar^®^ LD (Wako, Tokyo, Japan).

### Luciferase assay

JHOC-9 and OVISE cells were seeded in triplicates (or greater) in 96-well plates at densities of 1 × 10^4^ and 9 × 10^3^ cells, respectively. After 24 h, Lipofectamine^®^ 2000 Transfection Reagent (Thermo Fisher Scientific) was used to co-transfect cells with an pMIR control luciferase vector, pMIR wild-type CDH1 3′-UTR luciferase vector (CDH1 3′-UTR wt), or pMIR mutant-type CDH1 3′-UTR luciferase vector (CDH1 3′-UTR mut; addgene, Cambridge, MA, USA) and pMIR-REPORT^™^ β-gal Control Plasmid (Applied Biosystems). At 24 h of post-transfection, the activities of firefly luciferase and β-galactosidase were measured using the pMIR-REPORT^™^ miRNA Expression Reporter Vector System (Applied Biosystems), according to the manufacturer’s instructions. All experiments were performed in triplicates, and values are presented as means ± SDs.

### Statistical analysis

Cluster 3.0 (Stanford University, Palo Alto, CA, USA) was used to conduct the clustering analysis; Tree View 1.6 (Stanford University) was used for visualization. The calculated centered correlation distances and average linkages of tissue samples and genes were analyzed according to the ratios of individual abundance to the median abundance of all genes among all samples, as previously described [9]. Correlations between sample clusters and clinical parameters were analyzed using Fisher’s exact test.

Through a BRB-ArrayTools-based class comparison analysis, we used the *t*-test to identify genes with a differential expression among histological groups. Genes with a *p* value of <0.001 were considered statistically significant. Moreover, we performed a global test to determine the differences in expression profiles between classes by permuting the labels indicating the profiles that corresponded to various classes. The significance level of this test was defined as the proportion of permutations that yielded at least as many significant genes as did the actual data. The false discovery rate that was associated with a table row was defined as the estimated proportion of genes with univariate *p* values less than or equal to the gene representative of a false positive in that row.

For in vitro assays, the means of two independent groups were compared using a two-sided Student’s *t*-test. StatMate IV software (ATMS Co., Ltd., Tokyo, Japan) was used for statistical analyses, and a *p* value of <0.05 was considered to be statistically significant.

## Results

### Global miRNA gene expression profile in OCCC

**Fig 1:**
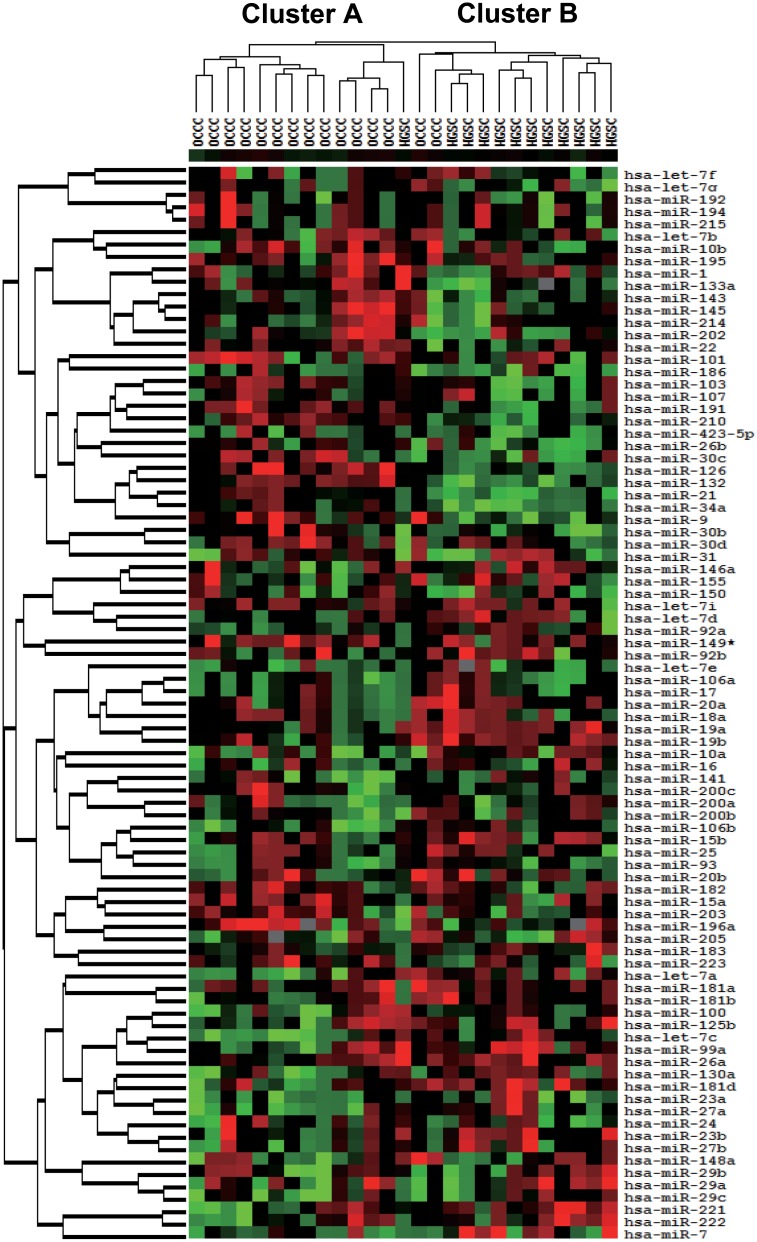
Cancer-related miRNA expression profiles of 27 patients with ovarian cancer. An unsupervised hierarchical clustering analysis of 87 miRNA expression in 27 patients with ovarian cancer, including the calculated centered correlation distances and average linkages, identified two main clusters, A and B.

We used the Cancer Focus microRNA PCR plate to survey the global expression of 88 miRNA genes including 85 cancer-related miRNAs that were identified from a large biobank and previous reports as either relevant oncogenes or tumor suppressors [10–15] in tumor specimens from 27 patients with ovarian cancer (S1 Data). A total of 87 miRNA expression values except *miR-206* in which the value was below the detectable threshold in the sample set, were used for subsequent analyses. An unsupervised hierarchical clustering analysis of these expression values classified these cancers with respect to similar expression patterns in the gene panel into two main clusters, A and B (Fig 1). Cluster A contained one of 13 HGSC cases and 15 of 17 OCCC cases; the remaining 12 HGSC and two OCCC cases comprised Cluster B. Statistically significant differences in the associations between clusters and clinicopathological parameters were observed with respect to histological type, FIGO stage, CA125 values, and endometriosis status; of these, histological type was the most significant (Table 2).

**Table 2:** Clinicopathological Characteristics of 27 Patients with Ovarian Cancer.

Parameters	Cluster A (n = 14)	Cluster B (n = 13)	*p*-value
**Patient age**			
≤60 years	12	11	1.000
>60 years	2	2	
**Histological type**			
OCCC	13	2	<0.0001*
HGSC	1	11	
**FIGO stage**			
I+II	12	6	0.046*
III+IV	2	7	
**LN metastasis (n = 26)**			
**No metastasis**	12	6	0.090
**Metastasis**	2	6	
**CA125 (n = 25)**			
**>35 U/ml**	8	12	0.039*
**≤35 U/ml**	5	0	
**Ascitic cytology (n = 26)**			
**Positive**	6	8	0.267
**Negative**	8	4	
**Rupture**			
**Without**	5	2	0.490
**Surgical**	2	3	
**Ruptured**	7	8	
**Endometriosis (n = 26)**			
**Positive**	10	3	0.026*
**Negative**	4	9	
**Thrombosis (n = 24)**			
**Positive**	1	1	0.537
**Negative**	12	10	

OCCC, ovarian clear cell carcinoma; HGSC, high-grade serous carcinoma; FIGO, International Federation of Gynecology and Obstetrics; LN, lymph node

*Significant difference

Next, we imported normalized expression data into BRB-ArrayTools and using a class comparison analysis, identified five miRNAs (*miR-132*, *miR-9*, *miR-126*, *miR-34a*, and *miR-21*) with a differential expression according to the histological type (Table 3). In particular, all five miRNAs were expressed at statistically higher levels in OCCC relative to HGSC in a univariate permutation test (p < 0.001).

**Table 3:** Differentially Expressed miRNAs in OCCC *vs*. HGSC.

miRNA	*p* value	FDR	Permutation *p* value	Fold change
*miR-132*	2.44e-05	0.00172	<1e-07	3.57
*miR-9*	7.31e-05	0.00172	<1e-07	8.11
*miR-126*	7.86e-05	0.00172	<1e-07	3.21
*miR-34a*	7.93e-05	0.00172	<1e-07	3.5
*miR-21*	0.00092	0.016	0.0012	3.44

OCCC, ovarian clear cell carcinoma; HGSC, high-grade serous carcinoma

A univariate analysis revealed that these five miRNAs were significant at a *p* value of <0.001. Permutation *p* values for significant genes were computed on the basis of 10000 random permutations.

### Validation of microRNA PCR plate data via individual real-time RT-PCR analysis

**Fig 2:**
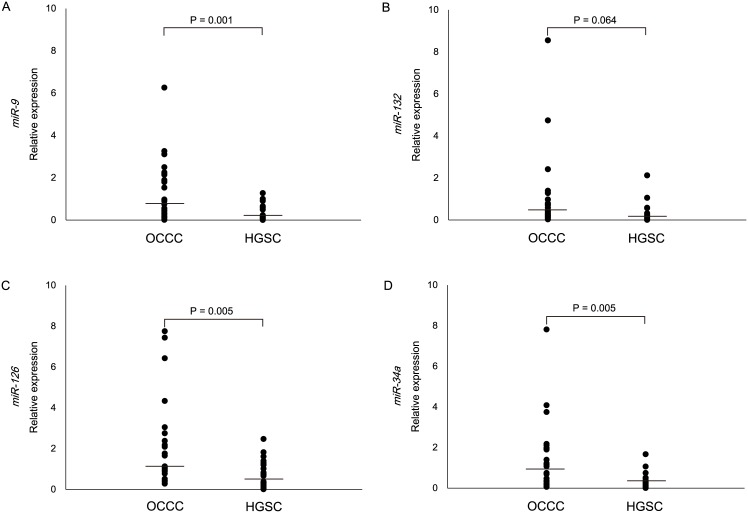
Validating original data by individual real-time RT-PCR analysis for both cohorts. Individual Taqman MicroRNA assays were used to analyze the relative expression of *miR-9* (A), *miR-132* (B), *miR-126* (C), and *miR-34a* (D) in the original 27 and additional 23 cases. Horizontal lines inside dots represent the median values.

Individual Taqman^®^ MicroRNA assays for *miR-9*, *miR-132*, *miR-126*, and *miR-34a* were used to validate the expression signatures that were determined via microRNA PCR plate analysis in the original 27 and additional 23 cases (Table 1). When we compared the relative expressions between OCCC and HGSC, the former expressed significantly higher levels of *miR-9*, *miR-126*, and *miR-34a* (Fig 2); of these, the most significant difference was observed for *miR-9*. In addition, *miR-132* expressions were clearly elevated in OCCC compared with HGSC, although this difference was only marginally significant (Fig 2).

### Involvement of *miR*-9 in OCCC pathogenesis

**Fig 3:**
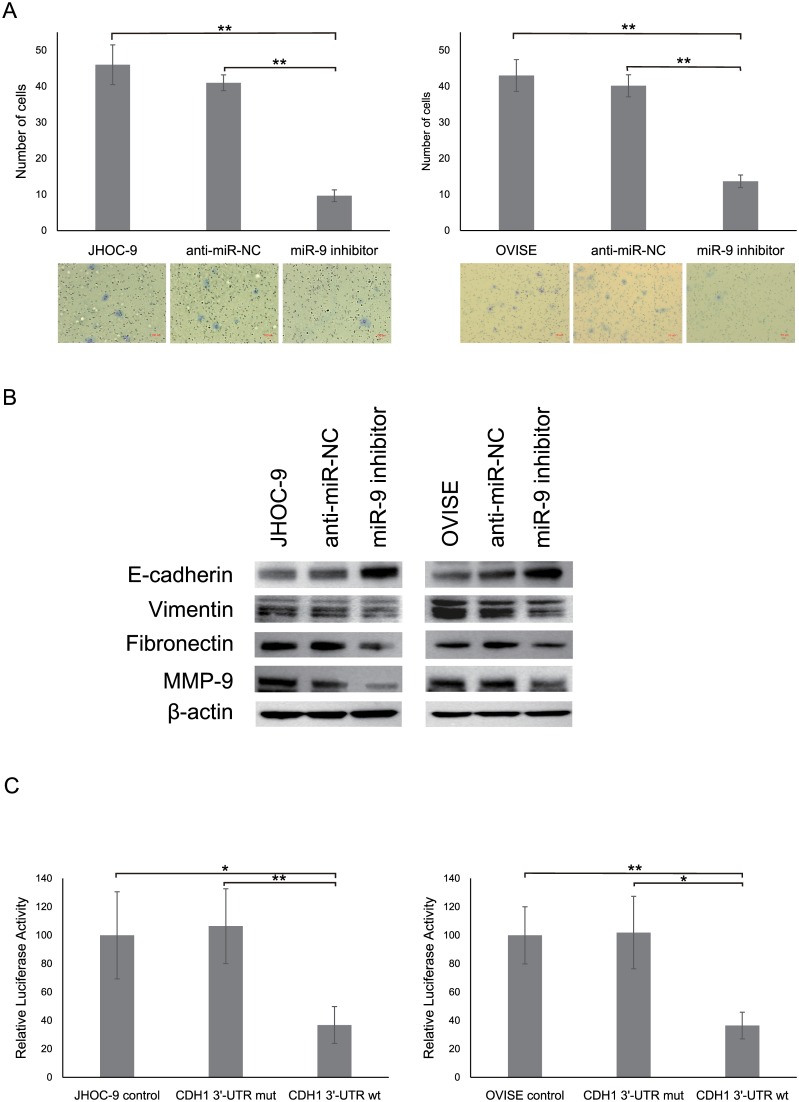
Effects of *miR-9* knockdown on biological responses of OCCC cells. (A) JHOC-9 and OVISE cells transfected with either a miR-9 inhibitor or anti-miR-NC were subjected to an invasion assay. All experiments were repeated at least three times, and values are presented as means ± SDs. **P < 0.001. Representative images are shown. (B) E-cadherin, Vimentin, Fibronectin, and MMP-9 protein expressions in JHOC-9 and OVISE cells that were transfected with either miR-9 inhibitor or anti-miR-NC were analyzed by Western blotting. β-Actin expression was used as a loading control. (C) A luciferase reporter assay using the pMIR-REPORT^™^ System was conducted to confirm direct binding of *miR-9* to *CDH1* (E-cadherin) 3′-UTR in JHOC-9 and OVISE cells. All experiments were repeated at least three times, and values are presented as means ± SDs. *P < 0.05, **P < 0.001.

Real-time RT-PCR analysis of 16 ovarian cancer cell lines revealed higher *miR-9* expression in OCCC cell lines compared with non-OCCC cell lines despite a lack of significant differences, these results were similar to those observed in clinical specimens (S1 Fig). To elucidate the biological role of *miR-9* overexpression in OCCC, we conducted miRNA inhibitor-based knockdown experiments in two OCCC cell lines (JHOC-9 and OVISE). Through in vitro invasion and migration analyses, we observed that in OCCC cells, despite similar proliferative capacities (S2A Fig), *miR-9* suppression significantly reduced the invasion and migration abilities compared with that in negative control or parental cells (Fig 3A, S2B Fig).

The results of previous studies [16–18] and web-based computational target prediction program assessments of *miR-9* prompted us to examine whether *miR-9* could target E-cadherin, a potential mediator of cell invasion. In JHOC-9 and OVISE cells, *miR-9* suppression significantly increased E-cadherin expressions (Fig 3B, S2C Fig) and was associated with reduced mesenchymal marker (e.g., Vimentin and Fibronectin) and matrix metalloproteinase 9 (MMP-9) expressions. To confirm the direct binding of *miR-9* to E-cadherin, pMIR luciferase vectors were transfected in JHOC-9 and OVISE cells, which were then subjected to a luciferase reporter assay. A significant decrease in relative luciferase activity was observed in cells that were transfected with CDH1 3′-UTR wt compared with cells transfected with CDH1 3′-UTR mut or control vector (Fig 3C). Altogether, these results indicate that E-cadherin is a direct target of *miR-9* in OCCC.

## Discussion

Numerous miRNA profiling studies have demonstrated an essential role of miRNAs in ovarian carcinogenesis with respect to different aspects such as histological subtype, clinical stage, prognosis, and chemoresistance [19, 20]. Consistent with previous studies, we used a global cancer-related miRNA expression analysis to identify unique tumor expression signatures that could reliably discriminate ovarian cancer histotypes. Of note, only approximately 80 cancer-related miRNAs from among >2500 human miRNAs could significantly differentiate the ovarian histological subtypes of OCCC and HGSC; of these, *miR-132*, *miR-9*, *miR-126*, *miR-34a*, and *miR-21* were the strongest classifiers. In other words, up- or downregulation of these miRNAs might affect the pathogenesis of these ovarian cancer subtypes. We previously reported a potentially important role for *miR-21* overexpression in OCCC oncogenesis by targeting of the *PTEN* tumor suppressor gene [21]. A miRNA microarray analysis recently identified that *miR-509* members and *miR-510*, which were not included in the microRNA PCR plate used in this study, could clearly distinguish OCCC from HGSC [22]. Similarly, low *miR-449* expression [23] and high *miR-30a*/30a* expression [24] were identified as characteristic of an OCCC-specific miRNA molecular profile. However, most of these reports lacked experimental verification of the biological significance with respect to OCCC molecular pathogenesis.

In this study, we observed high *miR-9* expression in OCCC, similar to previous studies describing many other types of cancer, including esophageal [16], breast [17], and colorectal cancer [18]. *miR-9* upregulation was found to facilitate metastasis in esophageal squamous cell carcinoma and breast cancer by inducing epithelial–mesenchymal transition (EMT) through direct targeting of E-cadherin [16, 17]. EMT is an important biological process by which epithelial cells undergo reduced cell–cell adhesion, lose cell polarity, and acquire motile and invasive properties, thereby contributing to cancer cell invasion and metastatic dissemination [25]. Notable features of EMT include reduced E-cadherin expression and increased both mesenchymal maker and MMP expressions. In OCCC cell lines used in this study, *miR-9* knockdown significantly reduced invasion and migration abilities while upregulating E-cadherin expression, suggesting that aberrant *miR-9* expression might play an important role in EMT activation in OCCC cells through direct binding to and subsequent downregulation of E-cadherin. E-cadherin expression could be a clinically relevant prognostic marker in OCCC patients [26, 27]. Although in vivo experiments with molecular analyses are needed to further refine the *miR-9* involvement in the development and progression of OCCC, it is possible that OCCC cells with *miR-9* overexpression could spread into the peritoneal cavity through the regulation of E-cadherin expression. Furthermore, the clinical implications of the association between *miR-9* and E-cadherin expressions should be clarified in a large-scale study of patients with OCCC. Recent observations have provided convincing evidence to suggest that *miR-200* family members (*miR-200a*, *miR-200b*, *miR-200c*, *miR-141*, and *miR-429*) and *miR-205* can activate EMT by targeting the EMT drivers ZEB1 and ZEB2 in various cancer types, including ovarian cancer [28–30]. In addition, *miR-181a* promotes TGF-β-mediated EMT via Smad7 repression in HGSC [31]. In contrast, *miR-506*, a new class of miRNA, suppresses EMT and metastasis in ovarian cancer by regulating both E-cadherin and Vimentin/N-cadherin [32]. Because EMT regulation may be an ideal therapeutic strategy with significant effects on patient prognosis and outcomes, modulating EMT-associated miRNAs in tumor cells should not be overlooked while investigating novel ovarian cancer treatments.

Data from miRNA target prediction databases and a literature search identified numerous tumor suppressive targets of the oncogenic *miR-9*, including FOXO1 in both non-small cell lung cancer [33] and endometrial cancer [34] and CDX2 in gastric cancer [35]. In contrast, tumor suppressive and/or chemosensitive effects of *miR-9* have been reported in HGSC [36, 37]. These findings have led to the hypothesis that *miR-9* may exert multiple functions in different cancers and in different histotypes of cancers in the same organ. Notably, *miR-9* targets other than E-cadherin may affect OCCC oncogenesis, thus warranting additional studies to explore possible roles for *miR-9* upregulation and define the molecular mechanisms involved in the pathogenesis of this specific type of ovarian cancer.

The precise mechanisms underlying aberrant *miR-9* expression in several cancers are not completely understood. A previous study proposed that activated forms of the MYC and MYCN oncoproteins promote *miR-9* expression via gene amplification in human cancers [17]. We recently demonstrated that in OCCC, a chromosomal amplification at 17q23-25 could lead to *miR-21* overexpression, which is located in the affected genomic region [21]. Notably, half of all miRNA genes are located in cancer-related genomic regions, including minimal regions of amplification in malignant tumors [2, 38]. In previous studies, we [39] and another research group [40] frequently observed chromosomal amplification at 1q22, which includes *miR-9*, in OCCC via comparative genomic hybridization analysis. Altogether, these findings support the idea that *miR-9* upregulation in OCCC could be explained in part by somatic copy number alterations.

In conclusion, *miR-9* upregulation may be involved in OCCC pathogenesis, a unique ovarian cancer subtype, by inducing EMT through E-cadherin modulation. Accordingly, *miR-9* may be a promising therapeutic target strategy for OCCC.

## Supporting Information

S1 Data Eighty-eight miRNA expression profiles in tumor specimens from 27 patients with ovarian cancer. (XLSX)

S1 Fig Relative expression of *miR-9* in ten OCCC cell lines and six non-OCCC cell lines. miRNA expression was quantified using the comparative method; *SNORD38B* snRNA was used as an endogenous control. Data are shown as means ± SDs.
(EPS)

S2 Fig Effects of *miR-9* knockdown on biological responses of OCCC cells. (A, B) JHOC-9 and OVISE cells transfected with either a miR-9 inhibitor or anti-miR-NC were subjected to a proliferative assay using the CellTiter 96 AQueous One Solution Cell Proliferation Assay kit (Promega, Madison, WI) following the manufacturer’s protocol (A) and a migration assay (B). All experiments were repeated at least three times, and values are presented as means ± SDs. *P < 0.05, **P < 0.001. (C) E-cadherin, Vimentin, Fibronectin, and MMP-9 protein expressions in JHOC-9 and OVISE cells that were transfected with either miR-9 inhibitor or anti-miR-NC were analyzed using Western blotting and shown as densitometric analyses. β-Actin expression was used as a loading control.
(EPS)
